# The incidence of un-indicated preoperative testing in a tertiary academic ambulatory center: a retrospective cohort study

**DOI:** 10.1186/s13741-015-0023-y

**Published:** 2015-12-15

**Authors:** Onyi C. Onuoha, Michael B. Hatch, Todd A. Miano, Lee A. Fleisher

**Affiliations:** Department of Anesthesiology and Critical Care, Perelman School of Medicine at the University of Pennsylvania, 3400 Spruce Street Dulles 680, Philadelphia, PA 19104 USA; Center for Clinical Epidemiology and Biostatistics, Perelman School of Medicine, University of Pennsylvania, Philadelphia, Pennsylvania USA; Department of Anesthesiology and Critical Care, Perelman School of Medicine, Senior Scholar, Leonard Davis Institute, University of Pennsylvania, Philadelphia, Pennsylvania USA

**Keywords:** Preoperative testing, Ambulatory, Low risk, Un-indicated, Routine, ASA (American Society of Anesthesiology), Laboratory test (complete blood count, metabolic panel, coagulation studies)

## Abstract

**Background:**

Despite existing evidence and guidelines advocating for appropriate risk stratification, ambulatory surgery in low-risk patients continues to be accompanied by a battery of routine tests prior to surgery. Using a single-center retrospective cohort study, we aimed to quantify the incidence of un-indicated preoperative testing in an academic ambulatory center by utilizing recommendations by the recently developed American Society of Anesthesiology (ASA) “Choosing Wisely” Top-5 list.

**Methods:**

We utilized data from the EPIC medical records of 3111 patients who had ambulatory surgery at the Hospital of the University of Pennsylvania during a 6-month period. Data were abstracted from laboratory studies— complete blood count, electrolyte panel, coagulation studies, and cardiac studies—stress test, and echocardiogram obtained within 30 days prior to surgery. Preoperative tests obtained from each patient were categorized into “indicated” (ASA ≥ 3) and “un-indicated” (ASA 1 and 2) tests, and percentages were reported.

**Results:**

During the study period, 52.9 % (95 % confidence interval (CI) 37.6–66.4) of all patients had at least one un-indicated laboratory test performed preoperatively. Further analysis revealed variation in the incidence of preoperative ordering between tests; 73 % of all complete blood counts (CBCs), 70 % of all metabolic panels, and 49 % of all coagulation studies were considered un-indicated by “Top-5 List” criteria. Stated differently, of the patients included in the sample, 51 % of patients received an un-indicated CBC, 41 % an un-indicated metabolic panel, and 16 % un-indicated coagulation studies. Twelve percent of “any un-indicated preoperative test” were obtained from ASA 1 healthy patients. Of the 587 patients less than 36 years old, 331 (56 %) had at least one test that was deemed un-indicated. Forty-one patients had either an echocardiogram or stress test ordered and performed within 30 days of surgery. Of these, eight (19.5 %) studies were un-indicated as determined by chart review.

**Conclusions:**

The incidence of ordering “at least one un-indicated preoperative test” in low-risk patients undergoing low-risk surgery remains high even in academic tertiary institutions. In the emerging era of optimizing patient safety and financial accountability, further studies are needed to better understand the problem of overuse while identifying modifiable attitudes and institutional influences on perioperative practices among all stakeholders involved. Such information would drive the development of feasible interventions.

## Background

With the release of the “Choosing Wisely” Top-5 lists of activities to avoid in 2013^1^ (Onuoha et al. 2014a), the American Society of Anesthesiologists (ASA) identified five diagnostic tests or treatments that are commonly practiced in the perioperative setting but offer limited to no benefits to patients according to evidence-based studies and may incur significant costs to the health system^1^ (Onuoha et al. 2014a; Onuoha et al. 2014b). Two of these items were preoperative recommendations focusing on unnecessary preoperative testing. They include the following:

Don’t obtain baseline laboratory studies in patients without significant systemic disease (ASA I or II) undergoing low-risk surgery - specifically complete blood count, basic or comprehensive metabolic panel, coagulation studies when blood loss (or fluid shifts) is/are expected to be minimal^1^, (Onuoha et al. 2014a; Onuoha et al. 2014b)

Don’t obtain baseline diagnostic cardiac testing (trans-thoracic/esophageal echocardiography – TTE/TEE) or cardiac stress testing in asymptomatic stable patients with known cardiac disease (e.g. CAD, valvular disease) undergoing low or moderate risk non-cardiac surgery^1^ (Austin et al. 2014; Benarroch-Gampel et al. 2012)

The ubiquitous use of routine testing in un-indicated patients has remained a hot topic for much over a decade (Benarroch-Gampel et al. 2012; Roizen 1997; Vogt and Henson 1997). In addition, the number of surgical procedures now performed on an outpatient basis continues to increase (Fleisher LA 2013; Richman 2010). It is estimated that about 30 million people undergo surgery annually in the USA, of which approximately 60–70 % are ambulatory procedures (Benarroch-Gampel et al. 2012; Fleisher LA 2013; Richman 2010). Ambulatory procedures are often performed in low-risk patients—healthy individuals or those with stable chronic medical conditions—and restricted to procedures of short duration with a low risk of intraoperative surgical complications (Benarroch-Gampel et al. 2012). Despite existing evidence-based guidelines advising the contrary, a battery of preoperative tests continue to be performed in low-risk patients undergoing low-risk ambulatory surgery (Benarroch-Gampel et al. 2012; Brown and Brown 2011; Fleisher LA 2013; Richman 2010; Schein et al. 2000; Soares Dde et al. 2013; Vogt and Henson 1997). Routine preoperative tests when performed in low-risk patients rarely change management and as much as 93 % of these tests are not indicated (Brown and Brown 2011). In a study by Benarroch-Gampel et al. (2012), the authors showed that although rates of testing were lower in patients with no comorbidities, rates remained high, with 54 % of patients receiving at least one preoperative test. The overall incidence of complications was less than 1 %, and after controlling for patient comorbidities and the operative procedure, neither testing nor the presence of abnormal results were associated with postoperative complications. With the combination of routine preoperative testing in the setting of an increasing prevalence of ambulatory surgery, the elimination of un-indicated tests in low-risk patients would promote patient safety, better quality of care, and result in substantial cost savings (Brown and Brown 2011; Fleisher LA 2013; Schein et al. 2000).

While most of the body of research driving evidence-based guidelines originate from academic tertiary institutions, it is not clear whether such institutions adhere to these guidelines, and hence, display a lower incidence of overuse of preoperative tests in low-risk patients undergoing ambulatory surgery than stated in the literature. To establish and quantify the incidence of the ordering of un-indicated preoperative tests in an academic tertiary ambulatory center, we conducted a retrospective cohort study of all patients who underwent outpatient surgery at the Perelman Center for Advanced Medicine (PCAM), Hospital of the University of Pennsylvania during a 6-month period.

## Methods

We obtained approval from the Institutional Review Board of the Perelman School of Medicine, University of Pennsylvania.

### Data sources

Data was abstracted from the EPIC^2^ medical records of 3918 patients who underwent ambulatory surgery at PCAM between the months of November 2012 and April 2013.

### Participants

We restricted our sample to patients scheduled for ambulatory surgery only in this dedicated facility. Ambulatory surgery was defined as a “same day or 23-hour-stay elective procedure.” Scheduled outpatient procedures upgraded to inpatient status due to intraoperative events were included in the study sample since unplanned intraoperative events have no effect on the initial preoperative testing decisions. In addition, we excluded procedures that used only local anesthesia or conscious sedation without an anesthesiologist or mid-level anesthesia provider, yielding a final cohort of 3111 patients.

### Study variables

- Surgical risk
  The preoperative period was defined as 30 days prior to the scheduled procedure. We defined all outpatient procedures taking place in the ambulatory setting as “low-risk surgery” as referenced in recommendation #1 of the Top-5 list (Onuoha et al. 2014a; Onuoha et al. 2014b). Preoperative patient and surgical characteristics were abstracted and included: age, gender, height, weight, surgical procedure performed, surgeon, surgical service/clinic, date of procedure, comorbidities, and ASA physical status score. We also obtained specific laboratory and imaging studies obtained within the 30-day preoperative period: complete blood count (CBC), metabolic panel (basic metabolic panel (BMP) or comprehensive metabolic panel (CMP)), coagulation studies (prothrombin time (PT), activated partial thromboplastin time (aPTT)) and cardiac studies (transesophageal/transthoracic echocardiography (TTE/TEE), stress test—exercise, persantine, dobutamine echocardiography).
- Patient health status
  Patient health status was defined using the ASA physical status (PS) score assigned by the clinical anesthesiologist on the day of surgery. Patients assigned ASA 1 or 2 were defined as patients “without significant systemic disease” as referenced in recommendation #1 of the “Top-5 List”^1^ (Onuoha et al. 2014a; Onuoha et al. 2014b). For the purpose of this study, significant systemic disease was defined as an ASA classification of 3 and above^3^ (Daabiss 2011; Hata and Moyers 2009; Vogt and Henson 1997).
- Defining “Indicated” vs “Un-indicated Testing”
  Preoperative BMP, CMP, and CBCs performed were categorized into “indicated” (obtained on a patient with ASA PS ≥3) and “un-indicated” (obtained on a patient with ASA PS <3). Coagulation studies were un-indicated if a patient was classified as ASA PS <3 *and* was not on any anticoagulant therapy. For cardiovascular function studies, a retrospective chart review was completed to establish the indication and rationale for the test performed within 30 days of the procedure. The review involved identifying both the ordering clinician and listed indications from related clinic notes, and reviewing the documentation of telephone encounters to further understand the rationale for the order placement. Of note, our data collection process through EPIC enabled us capture only studies ordered within the University of Pennsylvania Health System (UPHS). Hence, we were unable to capture radiographic studies ordered and performed outside UPHS.

### Data analysis

Our primary endpoint was the percentage of patients with *at least one* un-indicated laboratory test, in accordance with the previous literature (Katz et al. 2011), Katz et al. (2011) found that the number of inappropriate tests per patient follows a geometric distribution. The geometric distribution has a proportion (p) as its sole parameter. If the counts of un-indicated tests follow this distribution, knowing the percentage of patients with at least one un-indicated test provides just as much information as the number per patient (Katz et al. 2011). We examined this assumption with the chi-square goodness of fit test.

There are 12 surgical specialties that operate at PCAM ambulatory surgical center. We expected practice patterns to vary among the different specialties and the probability of un-indicated testing to be correlated within a specialty. We accounted for this correlation by using time series analysis (Dexter et al. 2005a; Dexter et al. 2005b). We tabulated the number of patients with at least one un-indicated test among successive batches of 4-week periods for each specialty and subsequently applied the Freeman-Tukey transformation to each of the *n* = 6 batches (Mosteller and Youtz 1961). Differences between specialties were examined using a one-way analysis of variance (ANOVA) on the mean of the transformed proportions (Austin et al. 2014). Confidence intervals (CIs) were calculated for each specialty using the Student 1-sample *t* test (Dexter et al. 2005a). We finally applied the inverse transformation to express the estimates as proportions (Dexter et al. 2005a). Five surgical specialties accounted for >80 % of all procedures performed. We collapsed the remaining surgical specialties into one category to avoid unstable estimates due to low numbers (Dexter et al. 2005a). We hypothesized the incidence of un-indicated testing to be ≥50 %.(Benarroch-Gampel et al. 2012; Katz et al. 2011; Mantha et al. 2005). We thus estimated the sample size required to obtain a lower bound of the 95 % CI ≥ 47 % to be 2915 patients. All data analyses were conducted using Stata/IC 12.1 for Mac (StataCorp, College Station, TX).

## Results

Patient characteristics are shown in Table 1. The majority of patients were female and classified as having “mild systemic disease.”

**Table 1:** Demographics: patient and surgical characteristics

Characteristic		Total *N* = 3111
		*N* (%)
Age years (range, mean, SD)	12.3 to 94.8	51.6 ± 16.5
Age (years)	≤35	587 (18.9)
	36—55	1153 (37.1)
	56—75	1143 (36.7)
	>75	228 (7.3)
Gender	Male	1106 (36)
	Female	2005 (64)
ASA physical status	1	348 (11.2)
	2	1972 (63.4)
	3	782 (25.1)
	4	9 (0.3)
Surgical specialties and incidence of un-indicated testing		
Surgical specialty	Frequency (percent)^c^	95 % confidence interval^b^
Endocrine oncologic	338/753 (55.1)	49.4–61.4
Gynecological	419/537 (78.0)	72.6–82.4
Otology	154/408 (37.5)	33.2–43.0
Plastic	199/408 (48.8)	43.3–53.3
Urology	220/467 (47.1)	41.1–53.9
Other^a^	241/538 (44.8)	42.2–48.0
All specialties	1648 /3111 (52.9)	37.6–66.4

Incidence of un-indicated testing among surgical specialties

^a^Colorectal, gastrointestinal, oral maxillofacial surgery, head and neck surgery, orthopedic, trauma, transplant

^b^Freeman-Tukey transformation among *n* = 6 batches of 4-week periods. Ninety-five percent confidence intervals calculated from the Student 1-sample *t* test among batches, with the inverse transformation taken

^c^Ordering rates were significantly different among specialties (Freeman-Tukey transformed ANOVA, *p* value = 0.001)

### Preoperative testing—laboratory (lab) data

**Fig. 1:**
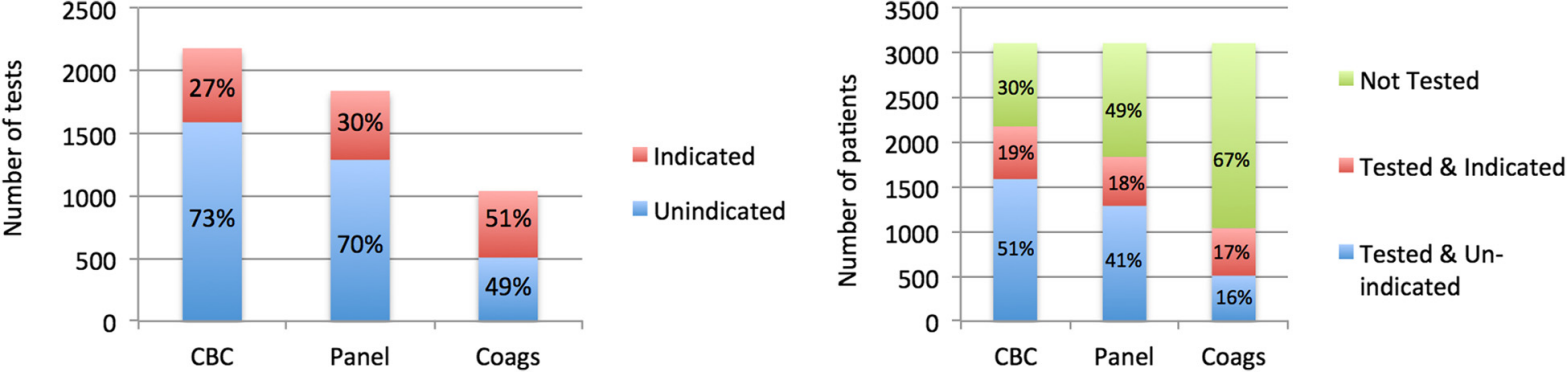
Profile of the incidence of un-indicated testing by test and by patient

**Fig. 2:**
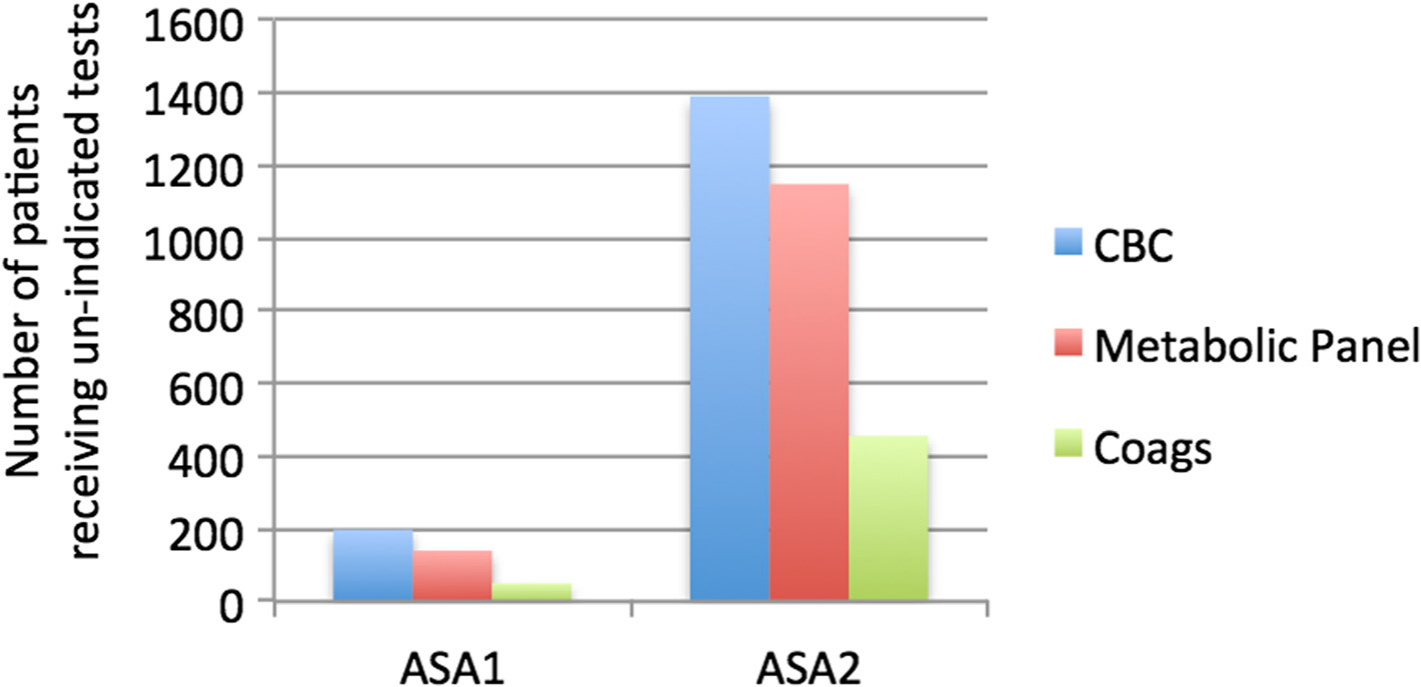
Profile of the incidence of un-indicated testing by ASA status. Most un-indicated labs are ordered in ASA 2 patients with CBC accounting for the most commonly ordered un-indicated lab test (1393 patients—45 % of the total patient population)

During the study period, 52.9 % (95 % CI 37.6–66.4) of all patients had at least one un-indicated lab test (CBC, metabolic panel, or coagulation study) performed preoperatively. The wide CI around this estimate is due to substantial heterogeneity in ordering across surgical specialties (Table 1). Further analysis revealed variation in the incidence of ordering between different tests. Seventy-three percent of all CBCs, 70 % of all metabolic panels, and 49 % of all coagulation studies were considered un-indicated. Stated differently, of the patients included in the sample, 51 % of patients obtained an un-indicated CBC, 41 % an un-indicated metabolic panel, and 16 % un-indicated coagulation studies (Fig. 1). In this cohort, 15 % (455) of the patients received all three laboratory test types and in each instance, the test was considered un-indicated. Of these 455 patients, 10 % were healthy ASA 1 patients. Un-indicated testing was present even among the youngest and healthiest of patients. Of the 587 patients less than 36 years old, 331 (56 %) had at least one test that was considered un-indicated and 12 % of patients with “any un-indicated preoperative test” were classified as ASA 1 patients (Fig. 2). Sixty-five percent of the orders were placed by a surgeon, 34 % by a nurse practitioner or physician assistant, and 1 % had no indicated ordering clinician.

### Geometric distribution

**Fig. 3:**
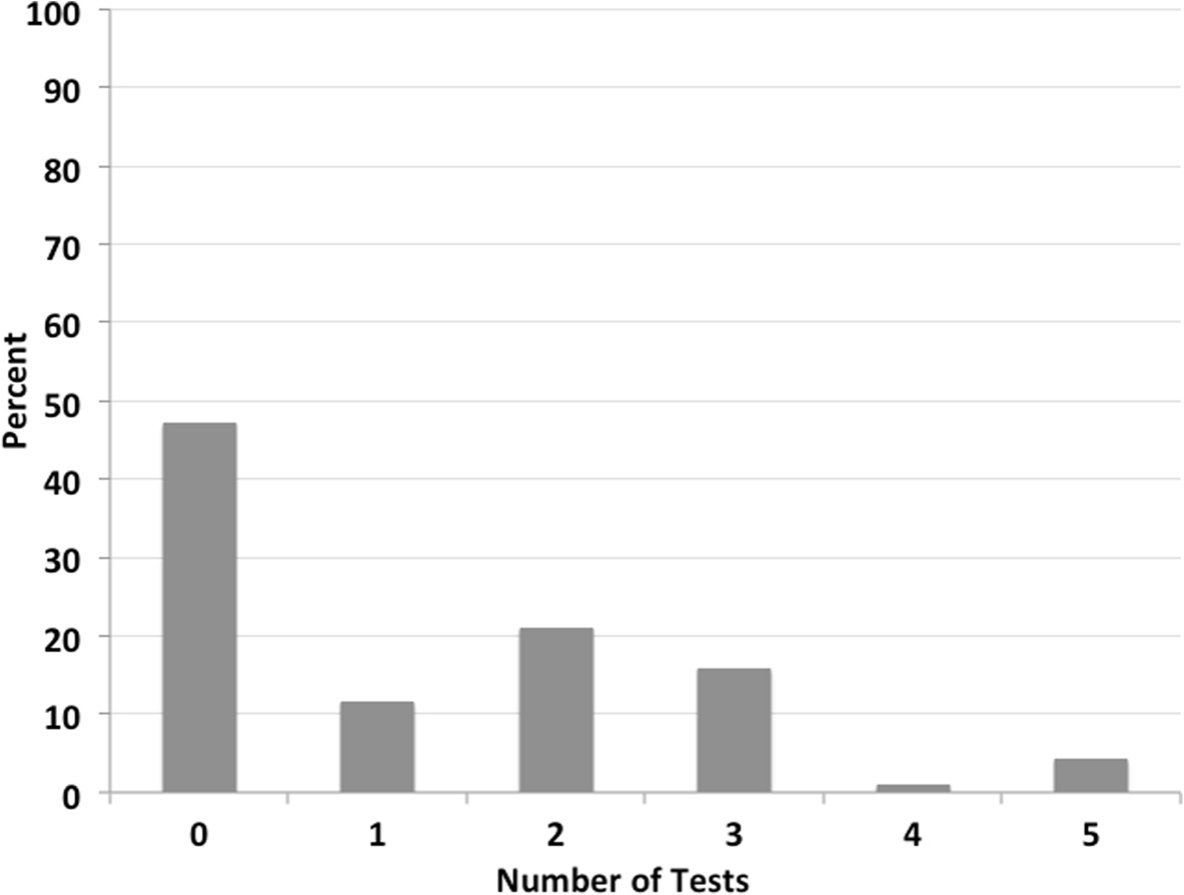
Number of un-indicated tests per patient

We found the distribution of the number of un-indicated tests per patient to depart significantly from the geometric distribution (*p* = 0.001, chi-square goodness of fit test). The departure was due to a lower-than-expected number of patients with only a single un-indicated test and a higher number of patients with two or more un-indicated tests (Fig. 3).

### Preoperative testing—cardiac imaging studies

Only 41 (1.3 %) patients in the sample had either an echocardiogram or stress test ordered and performed within 30 days of surgery. Of the 41 studies ordered within UPHS, 22 were ordered for reasons not related to surgery. For instance, 4 patients received surveillance echocardiograms for potentially cardiotoxic chemotherapeutic agents, 2 for an unrelated hospital admission, and 2 as surveillance studies for a history of a heart transplant. Of the 19 studies ordered for preoperative evaluation, a retrospective chart review revealed 11 were due to either known cardiac conditions deemed unstable by the ordering clinician, new electrocardiogram (EKG) findings or cardiovascular symptomatology in patients without preexisting cardiac disease. Eight studies were considered “un-indicated”—that is, they were ordered in the absence of cardiac disease or documented new cardiac symptoms.

## Discussion

Our study demonstrates a high incidence of obtaining “at least one un-indicated preoperative test” in low-risk patients undergoing ambulatory surgery despite multiple studies and guidelines (Committee on Standards and Practice Parameters et al. 2012; Czoski-Murray et al. 2012) addressing the lack of an indication for routine preoperative testing in this patient population. The issue of overuse transcends all types of practices and is pervasive even in the academic tertiary setting where most of the studies demonstrating the futility of low-value testing tend to be published. Our findings compare to other studies consistently showing a greater than 50 % risk of receiving at least one un-indicated laboratory test during preoperative evaluation (Benarroch-Gampel et al. 2012; Katz et al. 2011; Mantha et al. 2005).

During the past three decades, routine preoperative testing has been challenged by several academic publications with concerns about the sizeable cost of testing, false positive tests leading to unnecessary work-ups or treatments, and the unknown benefit of routine testing to patients (Kumar and Srivastava 2011). Obviously, the goal of preoperative testing should be to detect abnormalities that will alter management and ensure better patient outcomes (Benarroch-Gampel et al. 2012; Keay et al. 2012; Schein et al. 2000). However, several studies including randomized clinical trials continue to show no difference in outcomes when comparing routine to no preoperative testing (Benarroch-Gampel et al. 2012; Keay et al. 2012; Schein et al. 2000; Sheffield et al. 2013). Finding changes in tests of clinically healthy or stable patients usually does not alter clinical management during the perioperative period (Soares Dde et al. 2013). Instead, un-indicated investigations detect minor abnormalities of no clinical relevance which may be unsafe for patients causing unnecessary delay, further scrutiny of false positive or inconsequential findings, and cancellation of surgery and medico-legal liability if not addressed (Kumar and Srivastava 2011).

Onuoha et al. (2014b) in conjunction with the ASA conducted a survey of clinical anesthesiologists and results indicated that the utilization of low-value services are often driven by external factors other than patient safety such as the lack of control by anesthesiologists over preoperative testing, surgeon preference, patient preference or demand, medico-legal concerns, or postoperative needs. Additional predictors include facility preference, practice tradition, concerns about surgical delay or cancellation, institutional policies and procedures, and the lack of both clear guidelines or the awareness of current evidence with respect to preoperative testing (Benarroch-Gampel et al. 2012; Brown and Brown 2011; Soares Dde et al. 2013). In a survey of anesthesiologists, the most notable but modifiable challenge was the lack of communication and collaboration by all stakeholders involved in the perioperative care of the patient (Onuoha et al. 2014b).

### Clinical and research implications

With multiple studies establishing the persistent use of un-indicated preoperative testing, further studies are needed to not only identify modifiable attitudes and institutional influences on perioperative practices but also to develop and test feasible interventions that could curtail these practices. Most of the studies addressing preoperative testing originate in the anesthesia literature; however, approximately 80 % of preoperative tests are ordered by surgeons (Benarroch-Gampel et al. 2012; Onuoha et al. 2014b; Soares Dde et al. 2013). According to Soares Dde et al. (2013), when anesthesiologists take responsibility for preoperative tests, more appropriate tests are ordered via clinical profile, and consequently, surgery cancellations due to inadequate evaluation are reduced. Prior studies have also indicated a potential cost reduction of billions of dollars in preoperative testing without negatively affecting patient care when anesthesiologists assess patients and order tests prior to surgery (Fleisher 2000; Foss and Apfelbaum 2001; Soares Dde et al. 2013). Although preoperative clinics by anesthesiologists are effective (Foss and Apfelbaum 2001; Katz et al. 2011; Pollard 2002), many patients are not seen in them due to the unavailability of such clinics in several institutions. Hence, the effort to curtail un-indicated preoperative testing will require collaboration between anesthesia and surgical and primary care providers with associated mid-level providers, including nurse practitioners, nurse anesthetists, and physician assistants, to develop clinical pathways as to when preoperative tests are required. Increasing the awareness of the current evidence and guidelines through education of all departments and the institution of constant reminders in the electronic medical ordering system could be the first step. The creation and adherence to clear succinct evidence-based guidelines by a task force in the perioperative setting can be spearheaded by anesthesiologists and would at least begin to address the enforcement of existing practice parameters.

### Limitations of the study

Despite our findings, this study should be considered in the context of important limitations. First, the design of the study as a retrospective review makes it difficult to understand the decision making process when medical indications for preoperative testing are not documented clearly in the electronic medical record. Additionally, it is possible that testing may have been ordered as part of a diagnostic work-up of a presenting symptom rather than part of the preoperative screening process. We believe the contribution of error from this source to be negligible since over 90 % of the orders were placed as “outpatient orders.” Furthermore, as noted in the results, we found the distribution of the number of un-indicated tests per patient to depart significantly from the geometric distribution. Thus, in our institution, providers tend to order multiple un-indicated tests per patient, which suggests ordering is driven more by practice patterns than individual patient evaluation. Second, the use of the ASA PS classification as the sole measure of a patient’s health status may be an imperfect measure. In this study, an anesthesiologist assigned the ASA classification while the surgical staff placed the orders in question. Thus, the possibility exists that surgeons, in placing the preoperative screening orders, were considering factors in addition to those recognized by the anesthesiologist. Nevertheless, multiple studies including surveys by surgeons continue to show a routine instead of selective pattern to ordering preoperative tests (Benarroch-Gampel et al. 2012; Brown and Brown 2011; Schein et al. 2000; Soares Dde et al. 2013; Vogt and Henson 1997). A third limitation relates to the external validity and generalizability of our findings. PCAM at the Hospital of the University of Pennsylvania is a single-site institution and does not account for both the geographic or practice variability that can exist in other institutions. Involving multiple clinical sites in different parts of the country would provide better insight into the presence and enormity of this public health issue. These limitations notwithstanding, our findings carry important implications for current clinical practice, future research, and health policy in what is becoming an emerging era of optimizing patient safety and financial accountability. It also validates a pressing issue already described in several outpatient centers across the country.

## Conclusions

In summary, we demonstrated a high prevalence of ordering un-indicated preoperative tests in low-risk ambulatory surgery across multiple surgical specialties in an academic tertiary setting. Our findings emphasize the need for a collaborative effort among all perioperative providers to address this significant burden on the health care system. The creation and adherence to clear guidelines by a task force spearheaded by anesthesiologists would at least begin the process of implementing existing practice parameters.

## Abbreviations

ANOVA: analysis of variance
aPTT: activated partial thromboplastin time
ASA: American Society of Anesthesiologists
BMP: basic metabolic panel
CBC: complete blood count
CI: confidence interval
CMP: comprehensive metabolic panel
EKG: electrocardiogram
PCAM: Perelman Center for Advanced Medicine
PS: physical status
PT: prothrombin time
TEE: transesophageal echocardiography
TTE: tranthoracic echocardiography
UPHS: University of Pennsylvania Health System

## Footnotes

1 American Society of Anesthesiologists: Five things physicians and patients should question. Choosing Wisely: An initiative of the ABIM Foundation. Created by Onuoha OC, Arkoosh VA, Fleisher LA. 2013. http://www.choosingwisely.org/doctor-patient-lists/american-society-of-anesthesiologists/.

2 Epic Software. http://www.epic.com/software-index.php Accessed on 13 October, 2014.

3 American Society of Anesthesiologists: ASA Physical Status Classification System. https://www.asahq.org/resources/clinical-information/asa-physical-status-classification-system Accessed on 13 October, 2014.

## References

[CR1] Austin TM, Lam HV, Shin NS, Daily BJ, Dunn PF, Sandberg WS (2014). Elective change of surgeon during the OR day has an operationally negligible impact on turnover time. J Clin Anesth.

[CR2] Benarroch-Gampel J, Sheffield KM, Duncan CB, Brown KM, Han Y, Townsend CM (2012). Preoperative laboratory testing in patients undergoing elective, low-risk ambulatory surgery. Ann Surg.

[CR3] Brown SR, Brown J (2011). Why do physicians order unnecessary preoperative tests? A qualitative study. Fam Med.

[CR4] Apfelbaum JL, Connis RT, Nickinovich DG, Pasternak LR, Committee on Standards and Practice Parameters, American Society of Anesthesiologists Task Force on Preanesthesia Evaluation (2012). Practice advisory for preanesthesia evaluation: an updated report by the American Society of Anesthesiologists Task Force on preanesthesia evaluation. Anesthesiology.

[CR5] Czoski-Murray C, Lloyd JM, McCabe C, Claxton K, Oluboyede Y, Roberts J (2012). What is the value of routinely testing full blood count, electrolytes and urea, and pulmonary function test before elective surgery in patients with no apparent clinical indication and in subgroups of patients with common comorbidities: a systematic review of the clinical and cost-effective literature. Health Technol Assess.

[CR6] Daabiss M (2011). American Society of Anaesthesiologists physical status classification. Indian J Anaesth.

[CR7] Dexter F, Marcon E, Epstein RH, Ledolter J (2005). Validation of statistical methods to compare cancellation rates on the day of surgery. Anesth Analg.

[CR8] Dexter F, Epstein RH, Marcon E, Ledolter J (2005). Estimating the incidence of prolonged turnover times and delays by time of day. Anesthesiology.

[CR9] Fleisher LA (2000). Effect of preoperative evaluation and consultation on cost and outcome of surgical care. Current Opinion in Anesthesiology.

[CR10] Fleisher LA. Preoperative consultation before cataract surgery: are we choosing wisely or is this simply low-value care? JAMA Intern Med. 2014;174(3):389-90. jamainternalmedicine.com. 10.1001/jamainternmed.2013.12298 24366215

[CR11] Foss JF, Apfelbaum J (2001). Economics of preoperative evaluation clinics. Curr Opin Anaesthesiol.

[CR12] Hata TM, Moyers JR, Barash PG, Cullen BF, Stoelting RK, Cahalan MK, Stock MC (2009). Preoperative patient assessment and management. Clinical Anesthesia.

[CR13] Katz RI, Dexter F, Rosenfeld K, Wolfe L, Redmond V, Agarwal D (2011). Survey study of anesthesiologists’ and surgeons’ ordering of unnecessary preoperative laboratory tests. Anesth Analg.

[CR14] Keay L, Lindsley K, Tielsch J, Katz J, Schein O. Routine preoperative medical testing for cataract surgery. Cochrane Database Syst Rev. 2012;3:CD007293. doi: 10.1002/14651858.CD007293.pub3. 10.1002/14651858.CD007293.pub3 PMC4261928 22419323

[CR15] Kumar A, Srivastava U (2011). Role of routine laboratory investigations in preoperative evaluation. J Anaesthesiol Clin Pharmacol.

[CR16] Mantha S, Roizen MF, Madduri J, Rajender Y, Shanti Naidu K, Gayatri K (2005). Usefulness of routine preoperative testing: a prospective single observer study. J Clin Anesth.

[CR17] Mosteller F, Youtz C (1961). Tables of the Freeman-Tukey transformations for the binomial and Poisson distributions. Biometrika.

[CR18] Onuoha OC, Arkoosh VA, Fleisher LA (2014). “Choosing Wisely” in anesthesiology: Top-5 List—addressing the gap between evidence and practice. ASA Newsletter.

[CR19] Onuoha OC, Arkoosh VA, Fleisher LA (2014). Choosing wisely in anesthesiology: the gap between evidence and practice. JAMA Intern Med.

[CR20] Pollard JB (2002). Economic aspects of an anesthesia preoperative evaluation clinic. Curr Opin Anaesthesiol.

[CR21] Richman DC (2010). Ambulatory surgery: how much testing do we need?. Anesthesiol Clin.

[CR22] Roizen MF (1997). Preoperative evaluation: a shared vision for change. J Clin Anesth.

[CR23] Schein OD, Katz J, Bass EB, Tielsch JM, Lubomski LH, Feldman MA (2000). The value of routine preoperative medical testing before cataract surgery. N Engl J Med.

[CR24] Sheffield KM, McAdams PS, Benarroch-Gampel J, Goodwin JS, Boyd CA, Zhang D (2013). Overuse of preoperative cardiac stress testing in medicare patients undergoing elective noncardiac surgery. Ann Surg.

[CR25] Soares Dde S, Brandao RR, Mourao MR, Azevedo VL, Figueiredo AV, Trindade ES (2013). Relevance of routine testing in low risk patients undergoing minor and medium surgical procedures. Braz J Anesthesiol.

[CR26] Vogt AW, Henson LC (1997). Unindicated preoperative testing: ASA physical status and financial implications. J Clin Anesth.

